# Role of Toll Like Receptors in Irritable Bowel Syndrome: Differential Mucosal Immune Activation According to the Disease Subtype

**DOI:** 10.1371/journal.pone.0042777

**Published:** 2012-08-17

**Authors:** Liliana Belmonte, Stéphanie Beutheu Youmba, Nathalie Bertiaux-Vandaële, Michel Antonietti, Stéphane Lecleire, Alberto Zalar, Guillaume Gourcerol, Anne-Marie Leroi, Pierre Déchelotte, Moïse Coëffier, Philippe Ducrotté

**Affiliations:** 1 INSERM Unit U1073, Rouen University, Rouen, France; 2 Institute for Research and Innovation in Biomedicine, Rouen University, Rouen, France; 3 Laboratory of Immunology, IIHema, Consejo Nacional de Investigaciones Científicas y Técnicas, Academy National of Medicine, Buenos Aires, Argentine; 4 Gastroenterology Department, Rouen University Hospital, Rouen, France; 5 Physiology Department, Rouen University Hospital, Rouen, France; 6 Nutrition Unit, Rouen University Hospital, Rouen, France; Charité-University Medicine Berlin, Germany

## Abstract

**Background:**

The irritable bowel syndrome (IBS) is a functional gastrointestinal disorder whose pathogenesis is not completely understood. Its high prevalence and the considerable effects on quality of life make IBS a disease with high social cost. Recent studies suggest that low grade mucosal immune activation, increased intestinal permeability and the altered host-microbiota interactions that modulate innate immune response, contribute to the pathophysiology of IBS. However, the understanding of the precise molecular pathophysiology remains largely unknown.

**Methodology and Findings:**

In this study our objective was to evaluate the TLR expression as a key player in the innate immune response, in the colonic mucosa of IBS patients classified into the three main subtypes (with constipation, with diarrhea or mixed). TLR2 and TLR4 mRNA expression was assessed by real time RT-PCR while TLRs protein expression in intestinal epithelial cells was specifically assessed by flow cytometry and immunofluorescence. Mucosal inflammatory cytokine production was investigated by the multiplex technology. Here we report that the IBS-Mixed subgroup displayed a significant up-regulation of TLR2 and TLR4 in the colonic mucosa. Furthermore, these expressions were localized in the epithelial cells, opening new perspectives for a potential role of epithelial cells in host-immune interactions in IBS. In addition, the increased TLR expression in IBS-M patients elicited intracellular signaling pathways resulting in increased expression of the mucosal proinflammatory cytokines IL-8 and IL1β.

**Conclusions:**

Our results provide the first evidence of differential expression of TLR in IBS patients according to the disease subtype. These results offer further support that microflora plays a central role in the complex pathophysiology of IBS providing novel pharmacological targets for this chronic gastrointestinal disorder according to bowel habits.

## Introduction

Irritable bowel syndrome (IBS) is a common gastrointestinal disorder characterized by abdominal discomfort or pain and changes in bowel habits (constipation and/or diarrhea) [1], [2]. Although the exact origin of IBS symptoms remains unclear, a growing number of findings suggests that immune activation [3], [4], mucosal low grade inflammation [5], altered bacteria flora [6] and/or increased intestinal permeability [7], [8] play a major role in the pathophysiology of IBS. The intestinal epithelium serves as a barrier between the body and the microbiota and is an active agent in the mucosal immune response through its expression of pro-inflammatory genes, the secretion of inflammatory cytokines, and the recruitment of inflammatory cells in response to pathogenic bacteria and their products [9]. As a part of the innate immune response, pattern recognition receptors (PRRs) such as Toll-like (TLRs), NODs or NaLPs mediate this interaction between bacterial components, pathogen-associated molecular patterns (PAMPs) [10], [11], and the host. Human TLRs comprise a large family of 10 proteins [12] expressed on mucosal immune cells such as macrophages, or dendritic cells but also at low levels in the intestinal epithelial cells (IECs) [13]and bind specific molecules of diverse commensals and pathogens. Among TLRs, TLR2 and TLR4 have been the most extensively studied [10], [11]. TLR2 is required for the recognition of Gram-positive and mycobacteria components such as lipopeptide, lipotechoic acid, peptidoglycan, or soluble tuberculosis factor [14]. TLR4 and its co-receptors CD14 and LPS binding protein (LBP) are required for the recognition of lipopolysaccharides from gram-negative bacteria [10]. Interaction of PAMPs with TLRs elicits the activation of several transcription factors, particularly NF-κB and MAP kinases, and triggers pro-inflammatory cytokine production [15]. A dysregulated TLR signaling in intestinal epithelial cells (IECs) seems to be an important pathogenic factor in the onset of a chronic intestinal inflammation [16] and an inflammation-dependent induction of TLR2 and TLR4 expression in intestinal macrophages [17] has been recently reported. Furthermore, increased mucosal expression of TLR2, TLR3 and TLR4 was associated with inflammatory bowel disease [18], [19]. On the other hand, TLR activation is controlled by several negative regulators that include peroxisome proliferator-activated receptor-γ (PPARγ) [20]. PPARγ is a member of a nuclear receptor family highly expressed in the colonic epithelium and has the potential role of regulating colonic inflammation. Its activation inhibits the mucosal production of inflammatory cytokines [21] and an imbalance between elevated TLR4 levels and the expression of PPARγ in epithelial cells has been demonstrated in patients with ulcerative colitis [22].

Despite the fact that the TLRs are profoundly related with some gastrointestinal chronic inflammatory disorders, available data regarding their modulation in IBS pathophysiology are scarse. The present work therefore aimed at characterizing the expression and function of TLR2 and TLR4 by colonic mucosa of IBS patients, according to Rome III classification. In addition, we aimed to elucidate whether the TLR expression in the colon elicit intracellular signaling pathways resulting in the synthesis of proinflammatory cytokines to better define the pathogenetic role of TLR in IBS.

## Materials and Methods

### Study Population

Patients and controls were recruited in the Gastroenterology Department of the Rouen University Hospital. Consecutive individuals aged between 18 and 78 years who satisfied Rome III [2] criteria for the diagnosis of IBS were considered for inclusion in this study. Patients with organic gastrointestinal diseases, including inflammatory bowel disease, and clinically significant systemic diseases were excluded. The patients were classified in subtypes according to the Rome III criteria. Control subjects were recruited among the subjects admitted in the Gastroenterology Department for colorectal cancer screening or for follow-up after polyp removal. Among them, only those without any gastrointestinal symptom and normal colonoscopy were considered as healthy controls.

A complete colonoscopy was performed in all patients under general anaesthesia after large bowel cleansing with 4 liters of a macrogol solution. At least 10 biopsy specimens were taken in the descending colon in each IBS or control subject. Biopsy samples were immediately frozen then stored at −80°C until further processing. Two or three fresh additional biopsies from each subject were placed in cold PBS for cytometric analysis. Written consent to participate in the study was obtained in all cases. This study was approved by the Ethical Committee of the Rouen University Hospital (Comité de Protection des Personnes Nord-Ouest II), and complies with the International Declaration of Helsinki.

### Questionnaire

Study participants filled self-administered questionnaires to obtain demographic information and to describe their gastrointestinal symptoms and their psychological status. Questionnaire included questions about the onset, duration and evolution of IBS symptoms while the symptom severity was quantified with the validated Francis's score. Psychological well-being was assessed with the Hospital Anxiety and Depression scale (HAD).

### qRT-PCR

Mucosal total RNAs were extracted from colonic biopsies by a phenol-chloroform modified extraction method as described previously [23]. After reverse transcription of 1.5 µg total RNA into cDNA by using 200 units of SuperScript II Reverse Transcriptase (Invitrogen), qPCR for TLR2 and TLR4 was performed by SYBR Green technology on BioRad CFX96 real time PCR system (BioRad Laboratories, Marnes la Coquette, France) in duplicate for each sample. GAPDH was used as the endogenous reference gene. Specific primers were for *TLR2*, 5′ –TGATGCTGCCATTCTCATTC-3′ and 5′- CGCAGCTCTCAGATTTACCC-3′, for *TLR4*, 5′-CAGGGCTTTTCTGAGTCGTC-3′ and 5′-TGAGCAGTCGTGCTGGTATC-3′, and for *GAPDH*, 5′-TGCCATCAATGACCCCTTCA-3′ and 5′-TGACCTTGCCCACAGCCTTG-3′. Serially diluted cDNA samples were used as external standards. Absolute quantification of mRNA was performed by converting the sample cycle threshold (Ct) values to concentration (copies per ul) based on the standard curves. Sample TLR2 and TLR4 Ct were normalized relative to sample reference gene GAPDH.

### Flow cytometric analysis

Fresh biopsies were immediately placed in cold sterile PBS and processed within 2 h of endoscopical removal by mechanical disruption using scissors and passage over a mesh, using previously applied protocols [24]. The resulting mucosal suspension was evaluated by flow cytometry. The expression of surface markers on mucosal cells was analyzed using a FACSCalibur (BD Biosciences, San Diego, CA, USA) after staining with fluorochrome-conjugated mAbs: anti-human- EpCAM (Epithelial cell adhesion molecule, CD326, KSA, TROP1), -CD14 (61D3) purchased from eBioscience, -TLR2 (TL2.1), -TLR4 (HTA125) and isotype IgG controls, purchased from Imgenex (San Diego,CA). After staining, cells were washed with PBS and fixed with 1% paraformaldehyde.

For intracellular staining, cells were fixed and permeabilized with IC-Flow Kit (Imgenex, San Diego, CA) according to the instructions of the provider. After permeabilization, cells were stained with anti-human TLR4, same as above. The data were processed using Cell Quest software (BD Pharmingen) and FCS3 Express (DeNovo software). Acquisition of multiparameter data was carried out with an appropriate forward scatter (FSC) threshold to exclude debris. At least 10,000 intestinal epithelial cells per sample were analyzed as defined by forward and side scatter. Mean fluorescence intensity was calculated.

### Immunofluorescence

Fresh tissue samples were frozen in liquid nitrogen and stored at −80°C until further processing. Frozen tissue sections (10 µm thick) were obtained using a cryostat (Leica Microsystems) then were mounted on glass slides, and air dried. Nonspecific binding was blocked with PBS containing 1% bovine serum albumin (BSA, Sigma) or 10% normal goat serum (NGS) for 1 h at room temperature. Then, the sections were incubated at 4°C overnight in the same solution supplemented with primary antibodies mouse anti-TLR2 and anti-TLR4 (1∶1,000, Imgenex, San Diego,CA). After washes with PBS, sections were incubated with Rhodamine secondary Antibodies (Invitrogen) for 1 h at room temperature. Controls were assessed omitting the primary antibodies. Assessment of TLR2 and TLR4 protein labelling was performed by a single investigator (LB) who was uninformed of the patient group, on 10 light microscopy high-power fields using a ×200 lens.

### Mucosal cytokine assay

Protein extracts (50 µl) of colonic mucosa were processed in duplicate for concentrations of interleukin IL-1β, IL-6, IL-8, IFN-γ, and tumor necrosis factor- α (TNF-α) using a Fluorokine MAP kit (R & D Systems, Abingdon, UK). This assay relies on the use of polystyrene beads, each with a unique signature mix of fluorescent dyes that can be discriminated by a laser-based detection instrument, the Bioplex 2200 (BioRad Laboratories). Each bead type was coated with a specific antibody to the cytokine of interest. Results were expressed as pg/mg proteins.

### Western blot analysis

Biopsies were homogenized in ice-cold lysis buffer containing 0.1% protease inhibitor cocktail (Sigma Aldrich) as described previously [25]. Vials were placed on ice for 15 min and then centrifuged for 15 min at 4°C and 12,000 r.p.m. The supernatant containing proteins was collected and stored at −80°C until analysis. Proteins (25 µg) were separated on 4–12% Tris-Glycine resolving gels (Invitrogen, Cergy-Pontoise, France) and transferred to a nitrocellulose membrane (GE Healthcare, Orsay, France), which was blocked for 1 h at room temperature with 5% (w/v) non-fat dry milk in TBS (10 mmol/l Tris, pH 8; 150 mmol/l NaCl) plus 0.05% (w/v) Tween 20. Then, an overnight incubation at 4°C was done with monoclonal mouse anti-PPARγ (E8) antibodies (1∶1,000, Santa Cruz Biotechnologies, Inc) or with mouse anti-β-actin (1∶1,000, Sigma Aldrich) antibodies. After three washes in a blocking solution of 5% (w/v) non-fat dry milk in TBS/0.05% Tween 20, immunocomplexes were revealed by using the ECL detection system (GE Healthcare). Protein bands were quantified by densitometry using ImageScanner III and ImageQuant TL soft -ware (GE Healthcare).

### Statistical analysis

Statistical analysis was performed using GraphPad Prism 5.0 (GraphPad soft ware Inc, San Diego, CA). The nonparametric Kruskall-Wallis test, with Dunn's multiple comparison test, was used to evaluate differences between the 4 study groups. When only 2 groups were compared, a 2-tailed non parametric Mann-Whitney test was used. A nonparametric Spearman test was used to calculate correlations. r_s_: Spearman's correlation coefficient and its significance (p). For all tests, p<0.05 was considered significant.

## Results

### Clinical characteristics of patients

Forty eight consecutive IBS patients, 37 women and 11 men, mean aged 49±14 years and 31 control patients (17 women and 14 men, mean aged 57±14 years) were enrolled in this study. The IBS group consisted of 20 diarrhoea-predominant patients (IBS-D), 14 constipation-predominant (IBS-C) while 14 patients described a mixed bowel pattern (IBS-M) according to the Rome III definitions. In 6 patients, IBS was post-infectious while 23 patients reported a history of stressful life events. Mean Francis score was 273.4±96.4 (mean ± SEM). IBS patients had a mean HAD anxiety score of 10.5±4.3 and a mean HAD depression score of 5.8±3.6. Control patients did not match for gender and age. Thus, we checked in control patients that both age and gender did not affect the results (table 1).

**Table 1:** TLR2 and TLR4 expression in the colonia mucosa of control patients according to the gender and age.

	Gender		Age	
	*Male*	*Female*	*45.8±3*	*66.6±1.4*
**TLR2**	0.97 (0.03–5.6)	0.49 (0.05–4.28)	0.89 (0.03–4.28)	0.65 (0.05–5.6)
**TLR4**	0.47 (0.02–2.7)	0.54 (0.09–4.5)	1.03 (0.02–4.5)	0.46 (0.09–3.6)

Values are medians (range).

### Colonic TLR2 and TLR4 mRNA expression in IBS

**Figure 1:**
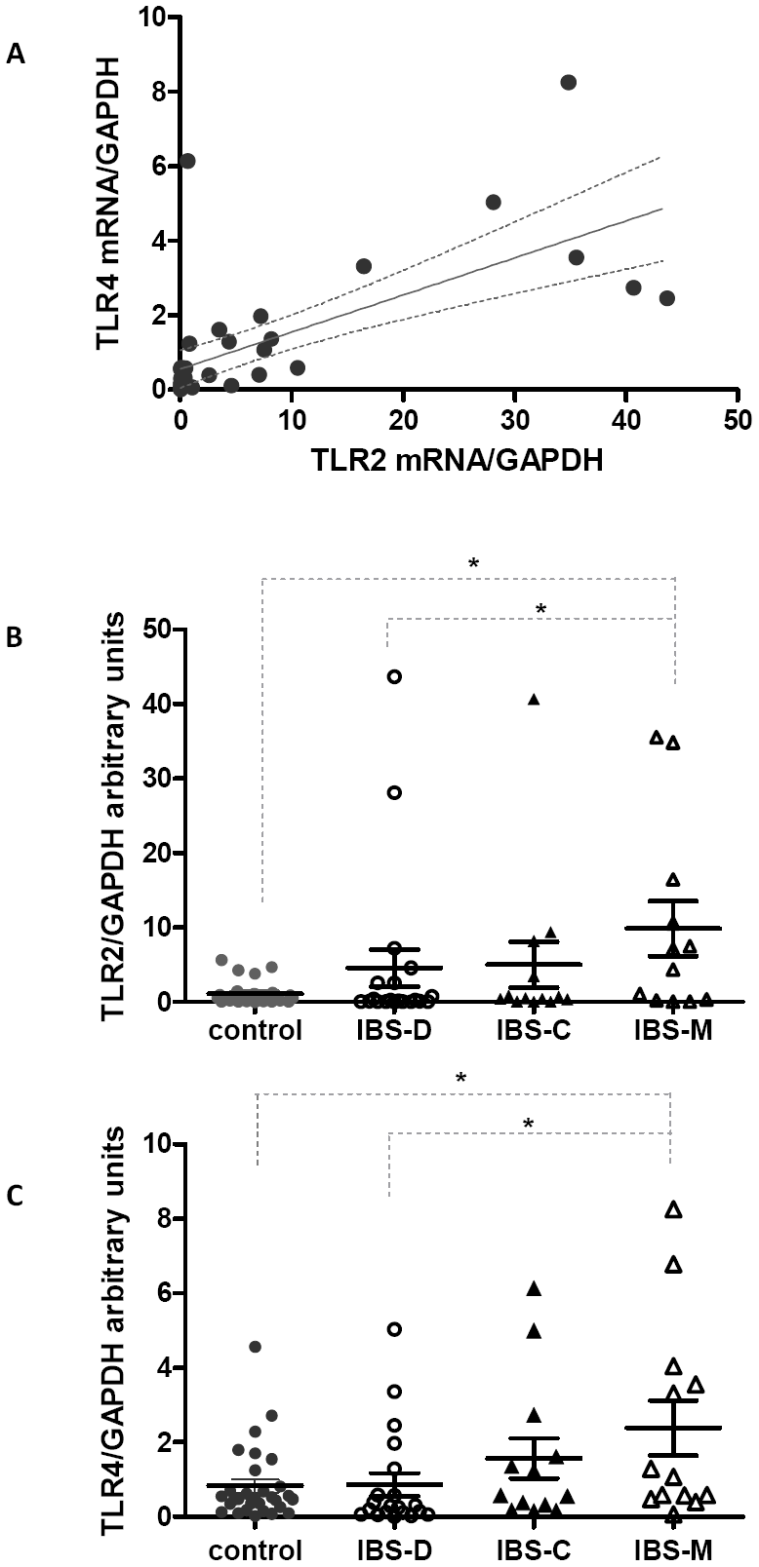
Expression levels of TLR2 and TLR4 in the colonic mucosa of controls and IBS patients. *(* ***A*** *)* Correlation between TLR2 and TLR4 mRNA expression in the whole group of IBS patients (r_s_ = 0.78, p<0.0001). *(* ***B*** *)* Changes in mRNA expression levels of TLR2 and *(* ***C*** *)* TLR4 in controls and in patients with predominant diarrhea (IBS-D), or constipation (IBS-C) or mixed (IBS-M) assessed by real-time PCR. Values are expressed as mean ± SEM. * Represents *p<0.05* using Mann-Whitney test.

When the whole IBS group was considered, there was no significant difference in the mean expression of TLR2 mRNA between IBS patients and controls (6.1±1.8 vs 1.9±0.6 respectively)(p = 0.46). These results were coincident with those of TLR4 mRNA expression, the relative expression value of TLR4 being 1.75±0.4 in IBS patients and 1.08±0.3 in controls (p = 0.2). In IBS patients, TLR2 and TLR4 mRNA values were strongly correlated (r_s_ = 0.78, p<0.0001) (figure 1A). When IBS subgroups were analyzed separately, TLR2 and TLR4 mRNA were differentially expressed between these sub-groups (p = 0.04 and p = 0.03 respectively). A significant seven-fold increase in the expression of TLR2 was detected in the IBS-M subgroup compared with controls (p = 0.02), while TLR2 values in IBS-M patients were also three-fold higher than that calculated in IBS-D and IBS-C patients (figure 1B).

The same tendency was observed for TLR4 gene expression. A significant two-fold increase in TLR4 expression was observed in IBS-M patients in comparison with controls and IBS-D patients (p = 0.04) (figure 1C).

### Correlation between colonic TLR2 and TLR4 mRNA levels with clinical parameters

**Figure 2:**
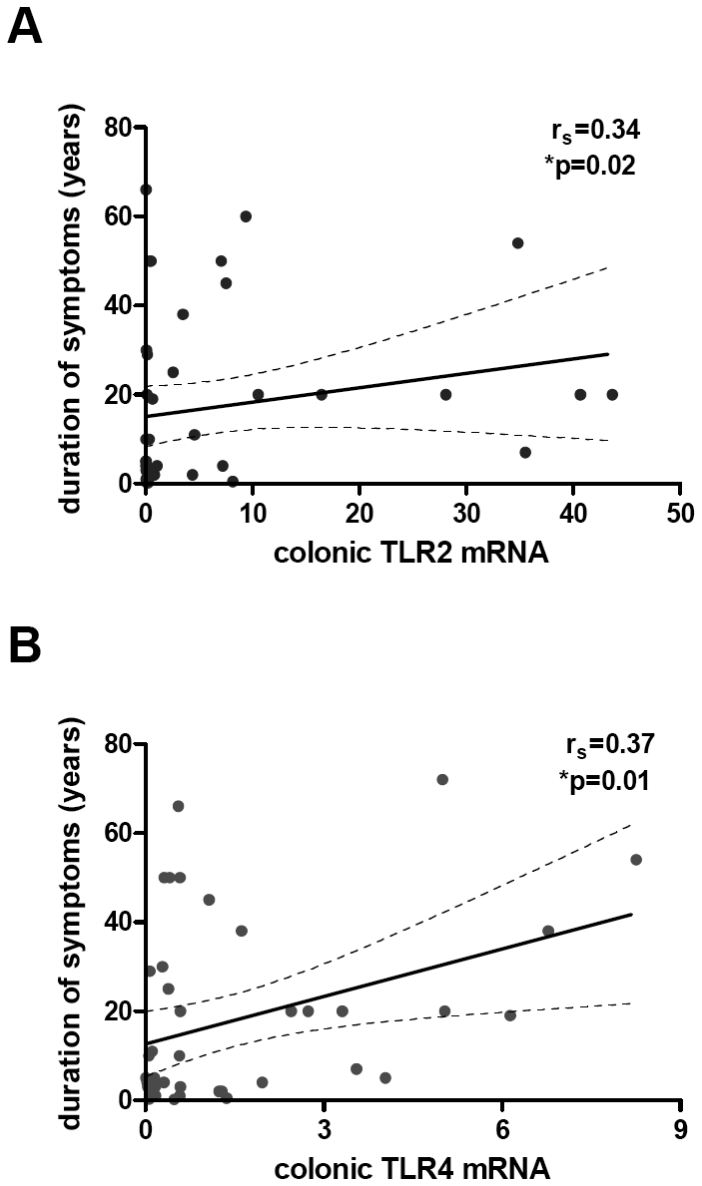
Correlation analysis of TLR2 and TLR4 mRNA levels and symptoms. Correlation between TLR2 *(* ***A*** *)* and TLR4 *(* ***B*** *)* expressions in the colonic mucosa and the duration of symptoms of IBS patients (n = 46, r_s_ = 0.34, p = 0.02 for TLR2 and r_s_ = 0.37, p = 0.01 for TLR4). Data were correlated by using non-parametric Spearman test.

TLR2 and TLR4 mRNA expression correlates significantly with duration of symptoms in the whole group of IBS patients (r_s_ = 0.34, p = 0.02 for TLR2 and r_s_ = 0.37, p = 0.01 for TLR4 (Figure 2A; 2B). However, when analysing the IBS subgroups, TLR2 expression was correlated to duration of symptoms in IBS-M patients (r_s_ = 0.56; *p = 0.03) but not in the other subgroups (Table 2). There was no correlation between duration of symptoms and TLR4 expression when IBS subgroups were analysed independently.

**Table 2:** Correlation analysis of TLR2 and TLR4 and duration of symptoms according to the subtype of IBS.

	IBS-D*n = 15*	IBS-C*n = 13*	IBS-M*n = 14*
**TLR2**	r_s_ = 0.03p = 0.91	r_s_ = 0.10p = 0.73	r_s_ = 0.56*p = 0.03
**TLR4**	r_s_ = −0.03p = 0.89	r_s_ = 0.36p = 0.22	r_s_ = 0.38p = 0.17

r_s_ and p values for Spearman correlation between TLR2 and TLR4 mRNA expression and duration of symptoms.

The highest TLR4 expression was observed in IBS patients who had symptoms lasting for more than 5 years (<5 years: 0.5±0.12 (n = 19) vs >5 years: 2.7±0.6 (N = 25),*p = 0.005).

In order to find out if TLR2 and TLR4 expression correlate with other clinical parameters, we analysed the Francis score's values. We did not find any association between TLR2 or TLR4 expression and Francis score (p = 0.85 for TLR2 and p = 0.46 for TLR4). No significant association was observed between TLRs expressions and the body mass index, the anxiety score or the depression score (data not shown).

### Expression of colonic TLR2 and TLR4 proteins in IBS

**Figure 3:**
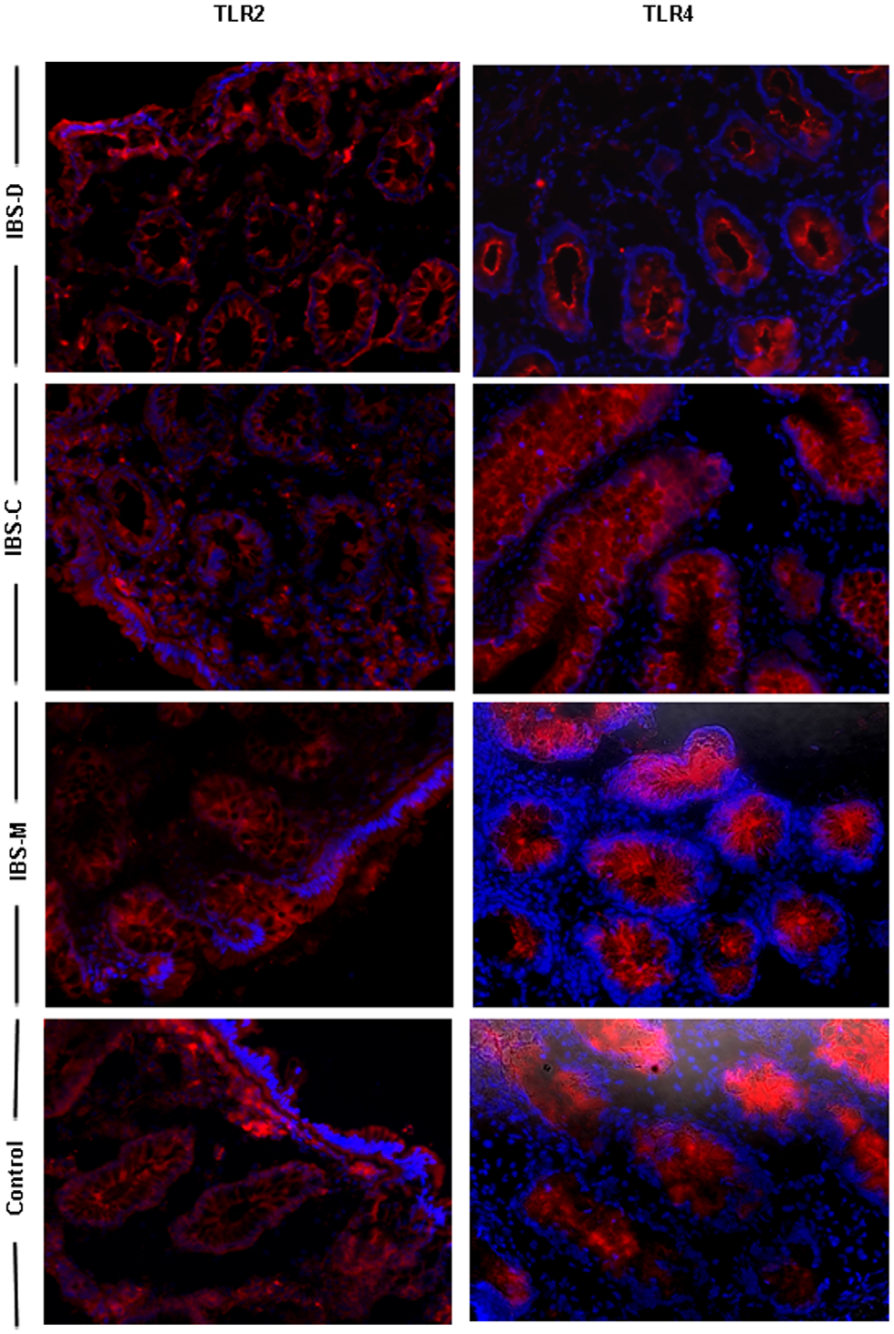
Immunofluorescence microscopy analysis of TLR2 and TLR4 in the colonic mucosa of IBS subgroups. Representative photomicrographs show the distribution of TLR2 and TLR4 proteins in the colonic mucosa of controls and IBS patients according to the disease subtype (IBS-Constipated, IBS-Diarrhea or IBS-Mixed alternating constipation with diarrhea) Red, TLR staining; blue, DAPI nuclear staining. Original magnification, ×20.

The mRNA analysis was performed on the whole colonic biopsies that may contain many cell types expressing TLRs. Therefore, to determine whether this mRNA expression pattern really reflected the expression of the TLR proteins, both immunofluorescence and flow cytometry were carried out. Immunofluorescence staining of colonic biopsies with Abs against TLR2 and TLR4 was performed to identify the cellular location of the receptors in IBS patients and controls. As shown in figure 3, TLR2 and 4 were principally expressed by IECs. TLRs expression was more intense in the crypts and less apparent in the surface epithelium when no expression of TLR2 and 4 were observed in the lamina propria.

**Figure 4:**
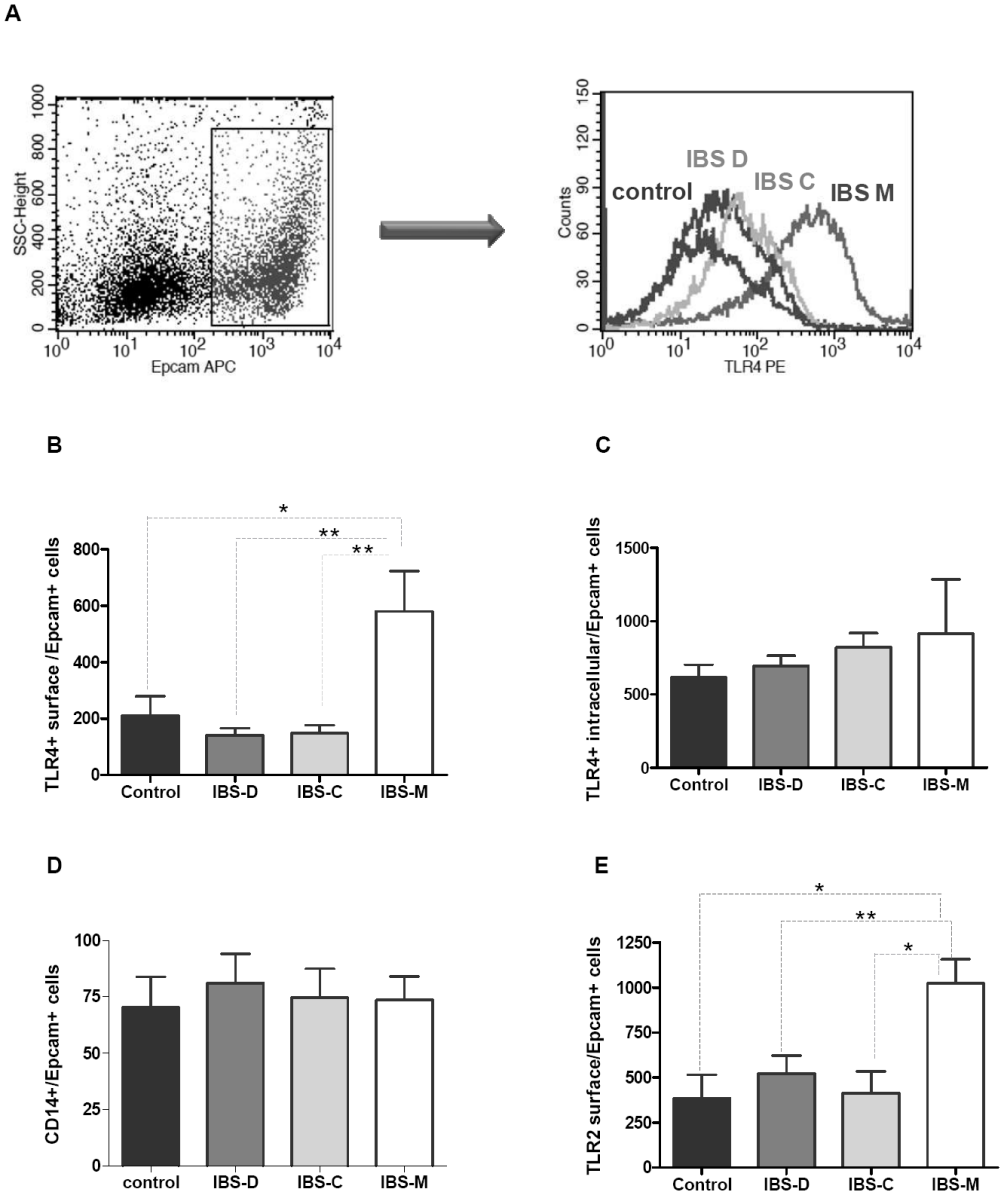
TLR2, TLR4 and CD14 expression in IECs (Epcam+ cells). Epithelials cells obtained from the colonic mucosa were assessed by flow cytometry as described in Patients and Methods. Mucosal colonic cells were double-stained with isotype IgG control or mAbs to TLR2, TLR4 or CD14 together with APC-conjugated anti Epcam mAbs. *(* ***A*** *)* Representative histogram showing the fluorescence intensity of surface TLR4 expression in gated Epcam+ cells from all IBS subtypes and controls patients. Changes in TLR4 surface expression *(* ***B*** *)*, intracellular TLR4 expression *(* ***C*** *)*, CD14 expression *(* ***D*** *)* and TLR2 surface expression *(* ***E*** *)* in IECs in control and IBS subtypes. Values are expressed as mean intensities fluorescence ± SEM. (*) Represents *p<0.05* and (**) represents p<0.005 using Mann-Whitney test. Kruskal-Wallis p values are *p = 0.01 (B); p = 0.5 (C); p = 0.95 (D) and *p = 0.03 (E) respectively.

In order to verify the presence of the TLRs proteins in IEC as well as to confirm the differences observed at the transcriptional level, Abs against TLR2 and TLR4 were used for flow cytometric analyses. Due to cell count limitations or occasional technical failure in tissue processing, this assay could not be performed for all patients. The study population comprised 10 IBS-D, 9 IBS-C, 7 IBS-M (mean age: 36.3 years) and eight control patients. We first investigated the cell surface expression of these receptors on IEC (Epcam+ cells) (figure 4A). No significant differences in the expression of TLR2 and TLR4 were observed in the surface of IECs, between the whole group of IBS patients and controls (data not shown); however, the fluorescence intensity of the TLRs varied markedly between the different IBS subtypes. TLR4 surface level was significantly increased in IBS-M patients compared to controls, which is in line with the real-time PCR results (figure 4B). When we examined the intracellular expression of TLR4 using permeabilized IECs, even if we detected intracellular localization of this receptor, no significant variations between IBS subtypes and controls were observed (figure 4C). As the co-receptor CD14 is essential for the response to LPS, we also evaluated its expression at the surface of IECs. No difference in its expression was observed between the different IBS subtypes and controls (figure 4D). The surface expression of TLR2 exhibited a similar pattern to that reported for TLR4 (figure 4E). The surface expression of TLR2 was significantly increased on IECs in IBS-M patients compared to controls and to IBS-C and IBS-D patients.

### Colonic expression of peroxisome proliferator-activated receptor gamma (PPARγ)

**Figure 5:**
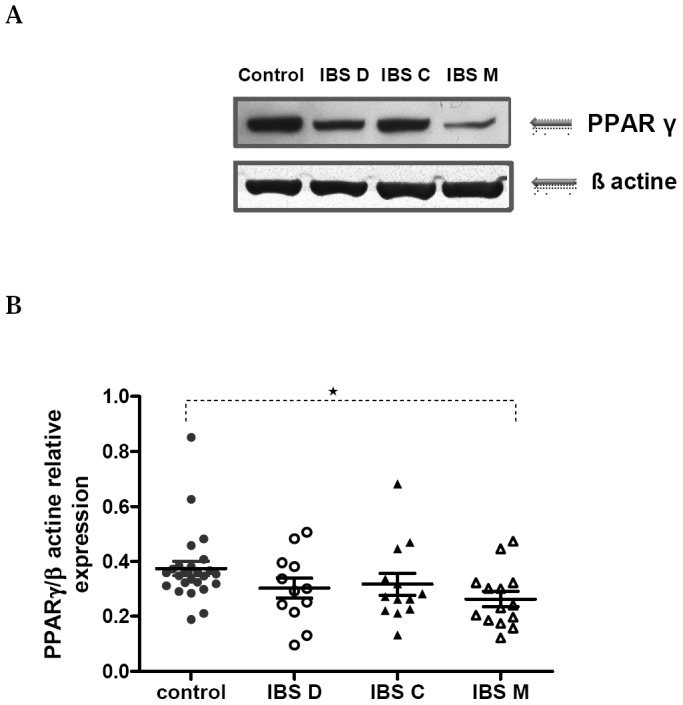
Relative PPARγ protein expression in colonic mucosa. *(* ***A*** *)* Representative western blot for PPARγ expression in colonic tissue from IBS subgroups and control. *(* ***B*** *)* Western blot analysis was performed for PPARγ expression on colonic biopsy lysates of control (n = 25) and IBS-C (n = 13), or IBS-D (n = 12), or IBS-M (n = 14). Data are expressed as means ± SEM; *p = 0.0033 vs. Controls, using Mann Whitney test.

Assuming the possible interaction between the pro-inflammatory TLR2 and 4 pathways and the anti-inflammatory PPARγ pathway, we investigated the expression of the negative regulator PPARγ in the colonic mucosa of IBS patients. As shown in figure 5, PPARγ protein levels were significantly lower in the colonic tissue in IBS-M patients than in controls (p = 0.003). In contrast, PPARγ levels were similar between IBS-D or IBS-C and controls.

### Mucosal cytokine concentrations

**Figure 6:**
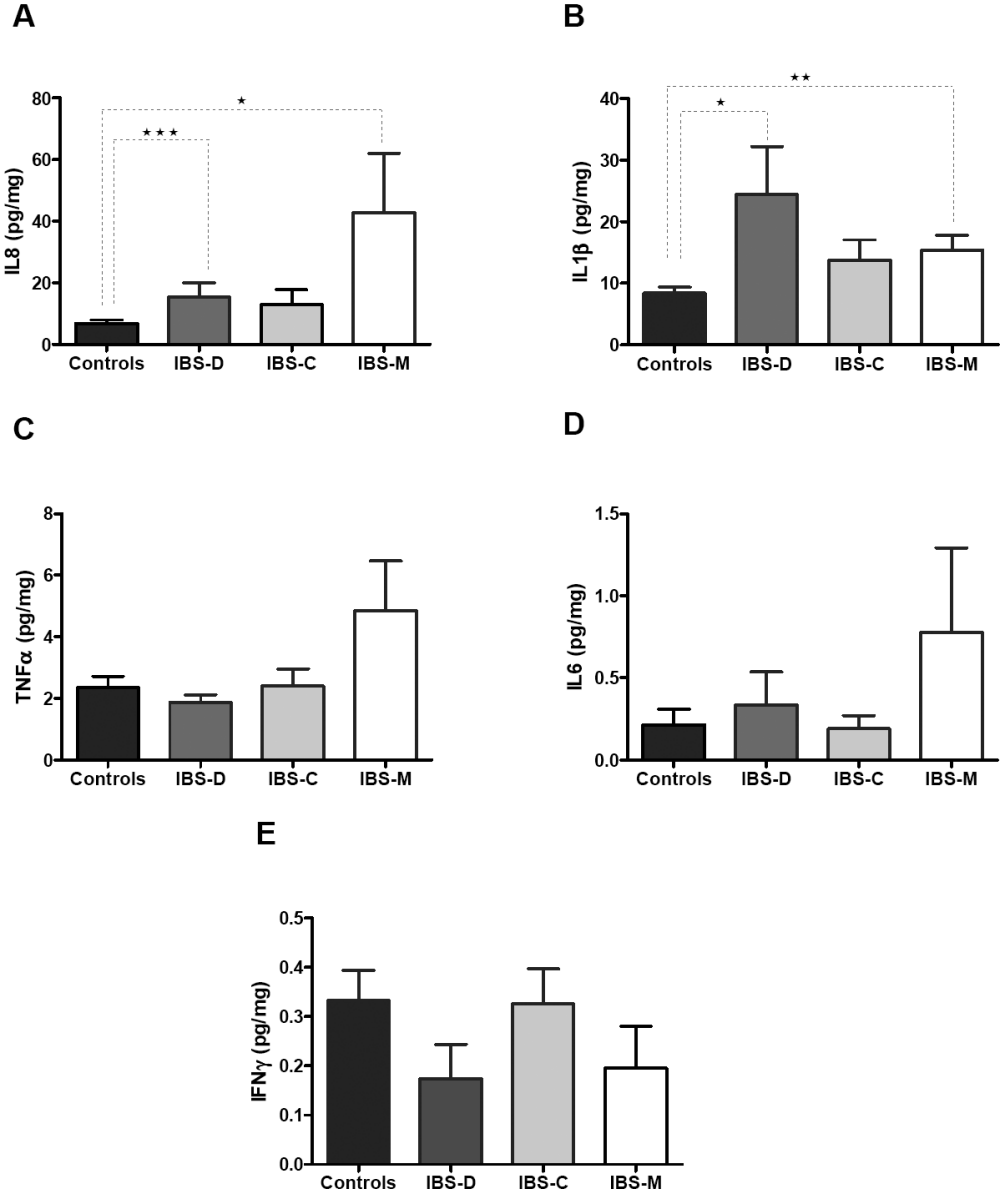
Levels of pro-inflammatory cytokines and chemokines in colonic mucosa. Cytokines were measured by multiplex cytokine bead immunoassays as described previously in Patients and Methods. Protein extracts of colonic mucosa were evaluated in duplicate for the expression of IL-8 *(* ***A*** *)*, IL1β *(* ***B*** *)*, TNF-α *(* ***C*** *)*, IL-6 *(* ***D*** *)* and IFN γ *(* ***E*** *)* in IBS patients and controls. Results were expressed as pg/mg protein. Differences in protein secretion were determined to be statistically significant by Mann-Whitney test with a value of p<0.05 (*), p<0.005 (**) and p<0.0005 (***).

Mucosal concentrations of pro-inflammatory cytokines and chemokines including TNF-α, IL-6, IL-1β, IL-8 and IFNγ were investigated. IL-8 and IL1β were significantly increased in IBS-M but also IBS-D patients compared to controls (figures 6 A and B). At variance, TNFα, IFNγ and IL-6 concentrations were not significantly different between controls and any IBS sub-type (figures 6 C, D, E).

## Discussion

Irritable bowel syndrome (IBS) is a functional gastrointestinal disorder characterized by abdominal pain and altered bowel habits in the absence of specific and unique organic pathology. IBS is common in the general population (worldwide prevalence of 10 to 15%) and has a significant medical and socioeconomic impact due to it changes the quality of life for the patients. Its pathophysiology is still not entirely clear and represents a research challenge. Emerging data suggest that a dysregulated intestinal immune response to microbiota might be involved in the pathophysiology of IBS, leading to an intestinal mucosal inflammation that sensitizes intestinal sensory endings [5]. In the present study, we have focused on the expression of TLRs in the colonic mucosa and we demonstrate that the colonic gene and protein expression of TLR2 and TLR4 differs significantly between the subgroups of IBS patients, providing further support for the hypothesis of altered intestinal immune activation.

The link between the activation of TLR2 and 4 and intestinal disease have been reported previously, both in the colon and the ileum of patients with inflammatory bowel diseases. TLR4 was strongly up-regulated in the intestinal epithelium of patients with both ulcerative colitis and Crohn's disease [18] in adults and children [19]. In the terminal ileum, a significant increase of TLR2 expression and an up-regulation of TLR4 have been reported in patients with active ulcerative colitis and Crohn's disease respectively [26]. Therefore, an abnormal immune response to microbiota is currently considered a relevant issue in elucidating the mechanisms underlying inflammatory bowel diseases.

Concerning IBS, while it is becoming clear that a low-grade inflammation may exist in the mucosal compartment, the triggering mechanisms of the relationship between the microbiota and the intestinal immune response remain to be completely elucidated. As the interaction between intestinal mucosa and microbes is partly mediated by TLRs, we consider that TLR activation and the subsequent inflammatory cytokines production in IBS needed to be investigated. Only two studies have already studied TLR expression in IBS. Brint et al have first reported, in the colonic mucosa of IBS patients, a four-fold and a 1.7 fold increase of TLR4 and TLR5 respectively while TLR7 and TLR8 expressions were 50 percent decreased when compared to controls [27]. These results were obtained in pooling data of different subtypes of IBS patients, without any sub-group analysis. In the second study, McKernann et al have described elevated cytokine levels and toll-like receptor activity in the blood and not at the mucosal levels in IBS patients [28].

However, to our knowledge, our study is the first in analysing the colonic gene and protein expression of TLR2 and TLR4 in the IBS subgroups. We describe an unexpected finding of a significant increase of TLR2 and TLR4 only in IBS-M subgroup compared with healthy subjects. These results support the hypothesis, at least in IBS-M patients, that the innate immune system plays a key role in the pathophysiology of the disease. The increased expression of TLRs was not established for the whole group of IBS when compared to controls, which does not support the recent report of Brint et al [27]. These differences may be due to the well-known heterogeneity of the IBS population. However, we have found a strong correlation between TLR2 and TLR4 mRNA (figure 1A), confirming that distinct TLRs synergize for optimal stimulation of innate immune system in the gut in response to microflora [29]. Therefore, both sensing of Gram-positive and Gram-negative bacteria by TLR2 and TLR4, respectively could result in immune system activation and secretion of proinflammatory cytokines [30].

With regard to the clinical relevance of these findings, we found a positive correlation between TLR2 and TLR4 expression and the duration of symptoms in the whole group of IBS patients (figure 2). However, when we analyzed the results according to IBS subgroups, a significant correlation was found only in IBS-M (table 2). Further studies will be necessary to confirm these results in the other subgroups of IBS. The mechanisms underlying the increasing expression of TLRs during the course of the disease remain unknown but we could assume that luminal factors may be involved. Very little information is at present available regarding how PAMPs concentrations in the intestinal contents may be altered during the course of the IBS altering the expression of TLRs.

An important issue in this study is the identification of the IBS-M subgroup. Its classification remains a clinical problem and, in our study, colonic biopsies were taken in these patients when transit disturbances were either diarrhea or constipation at the time of colonoscopy. Nevertheless, we considered those patients as IBS-M patients according to the clinical definition of IBS-M based on the Rome III criteria, and in all patients of our series, the disease duration was longer than one year that is a suitable criteria for a relevant clinical definition according to recent Drossman's recommendations [31].

On the other hand, there is also a need to clarify the cellular elements expressing TLR in the colon. Our immunostaining experiments demonstrate that TLR2 and 4 were present in the crypts and luminal surface and we could also localize its expression in epithelial cells, opening new perspectives for a potential role of epithelial cells in host-immune interactions in IBS. The protein expression profile of TLR2 and TLR4 on colonic epithelial cells (EpCam+ cells) assessed by flow cytometry demonstrated enhanced expression of these two receptors in the surface of EpCam+ cells of IBS-M patients. Thus, it seems that both TLR2 and TLR4 mediate signaling at the cell surface of the responding cell in this group of patients. Whether the increased expression of these receptors is observed only in IBS-M needs further investigation. If such changes are the cause or the consequence of an altered microbiota in these patients or only the result of constipation and diarrhea alternance is unclear. It has been established from comparative studies of germ-free and colonized animals that the microbiota influence the structure and immunological function of the gastrointestinal tract [32]. Previous studies suggested that fecal microbiota is significantly altered in IBS, and the microbial composition also differs among patients with diarrhea-predominant, constipation-predominant, and mixed types of the syndrome. Kassinen et al have reported that IBS-M was characterized especially by Bacteroides and Allisonella sequences [6]. These results support the hypothesis of specific pathophysiological changes in the IBS-M sub-group.

In this study, we also tried to investigate in depth how this up-regulation of TLR2 and TLR4 could promote an increased cytokine production in IBS-M patients. Among the various signalling proteins involved in regulating TLR-mediated gene expression, PPARγ deserved a particular attention as a potential inhibitor of colonic inflammation. This nuclear receptor is highly expressed in colonic epithelium [21] and in immune cells within the gut mucosa and is implicated in modulating inflammation and immune responses. Using Western blot on colonic biopsy samples of patients with IBS and controls, we observed an impaired expression of PPARγ in patients with IBS-M. The imbalance between elevated levels of TLR4 and the impaired expression of PPARγ suggests an altered response to luminal bacteria leading to colonic inflammation.

Even if one may argue that the assessment of TLR expression would be of greater importance in the right colon or in the terminal ileum, at the site of highest concentrations of bacteria and where most immunological engagement occurs, our data are consistent with the concept that an innate immune activation occurs in at least a subgroup of patients with IBS. The interaction between the TLRs to induce cellular activation and the mechanisms by which this event can affect the pathophysiology of IBS-M, also merit future research.

In conclusion, in IBS-M patients, an increased colonic expression of TLR2 and TLR4 is observed, accompanied by impaired expression of PPARγ and enhanced production of mucosal pro inflammatory cytokines. Evidence for dysbiosis in IBS has been reported [6], although, it is unclear, whether this event can be the cause or the consequence of the high levels of TLR2 and TLR4 observed in the colonic epithelium in this group of patients. Further studies about the composition of the host microflora in IBS subgroups will be necessary to understand its accurate implication in intestinal inflammation.
